# Aberrations Involving Chromosome 1 as a Possible Predictor of Odds Ratio for Colon Cancer - Results from the Krakow Case-Control Study

**DOI:** 10.1371/journal.pone.0147658

**Published:** 2016-01-29

**Authors:** Aleksander Galas, Justyna Miszczyk

**Affiliations:** 1 Department of Epidemiology, Jagiellonian University–Medical College, Krakow, Poland; 2 Department of Experimental Physics of Complex Systems, Institute of Nuclear Physics, Polish Academy of Sciences, Krakow, Poland; University Medical Center of Princeton/Rutgers Robert Wood Johnson Medical School, UNITED STATES

## Abstract

**Background:**

There is still an open question how to predict colorectal cancer risk before any morphological changes appear in the colon.

**Objective:**

The purpose was to investigate aberrations in chromosomes 1, 2 and 4 in peripheral blood lymphocytes analyzed by fluorescence *in situ* hybridization technique as a tool to assess the likelihood of colorectal cancer.

**Methods:**

A hospital-based case-control study included 20 colon cancer patients and 18 hospital-based controls. Information about potential covariates was collected by interview. The frequency of stable and unstable chromosome aberrations in chromosome 1, 2 and 4 was assessed by fluorescence *in situ* hybridization technique.

**Results:**

Colorectal cancer patients, as compared to controls, had a relatively higher frequency of chromosome 1 translocations (median: 3.5 versus 1.0 /1000 cells, p = 0.006), stable aberrations (3.8 versus 1.0 /1000 cells, p = 0.007) and total aberrations (p = 0.009). There were no differences observed for chromosomes 2 and 4. Our results showed an increase in the odds of having colon cancer by about 50–80% associated with an increase by 1/1000 cells in the number of chromosome 1 aberrations.

**Conclusions:**

The results revealed that the frequency of chromosomal aberrations, especially translocations in chromosome 1, seems to be a promising method to show a colon cancer risk. Additionally, our study suggests the reasonableness of use of biomarkers such as chromosome 1 aberrations in peripheral blood lymphocytes in screening prevention programs for individuals at higher colon cancer risk to identify those who are at increased risk and require more frequent investigations, e.g. by sigmoidoscopy.

## Introduction

Colorectal cancer (CRC) is among the top three most frequent cancers diagnosed in high-income countries and in the majority of middle-income countries. It also contributes to the high amount of cancer deaths worldwide. There is a large body of knowledge with respect to risk factors for CRC and majority of them point to the role of lifestyle as a main determinant, although several studies failed to prove a causal relationship [1]. Genetic predisposition has been estimated to contribute for about 35% of CRC cases [2]. The majority of studies which tried to assess changes responsible for development of colorectal cancer focused on genetic features observed in cancer tissue. Mutations in the adenomatous polyposis coli (APC) gene, MLH1, MSH2, PMS2 and MSH6 mismatch repair genes, and CpG island methylator phenotype (CIMP) have been recognized and well described [3]. Additionally, the involvement of TP53, TGFB1, and the mitogen-activated protein kinase (MAPK) signaling pathways were found to lead to the progression of CRC due to a loss of cell growth and differentiation control, and apoptosis [3,4]. Genome wide association studies (GWAS) carried out since 2007 have failed to find a common genetic variant responsible for the development of sporadic colorectal cancer. Even if some of those studies have found some single nucleotide polymorphisms (SNPs), they did not prove a causal relationship and the observed associated increase in CRC risk was only modest (an increase by 5% to 30%) [5]. There is an evidence that shows an increased risk for hereditary CRC among first relatives [6], but little is known about the predicting the risk for developing sporadic colorectal cancer among healthy individuals who do not have family history of CRC.

CRC develops as a consequence of local tissue changes [7], but there is still an open question on how to predict cancer risk before any morphological changes appear in the colon. It is for this reason we have investigated the odds of CRC associated with chromosomal damage measured in peripheral blood lymphocytes (PBL).

There is currently no established chromosome risk assessment marker for CRC. One recent study, published in 2013 by Chen [8], has shown that a majority of hypermethylated genes are suspected to be an epigenetic event that silences the tumor suppressor genes in CRC. These genes are placed on chromosome 1. Results from another study of eighty-three CRC patients suggested 1q31.3–32.1 (EEF1AL12) to be the region which might harbor at least one CRC tumor suppressor gene [9]. Xiao et al. investigated common chromosomal alterations in sporadic CRC and have found chromosomes 1, 2 and 4 to have chromosomal gains [10]. All these findings, although promising, have been observed as a difference between healthy and cancerous tissues, which limits their utility as a predictive biomarker for assessing risk of developing CRC. Considering cancer as the progressive accumulation of DNA damage during the deregulation of cellular processes, these results are correlative and do not discriminate the relationship on the basis of observed genetic changes as causal or causative, especially given the temporal and spacial heterogeneity of genetic changes present in CRC [11].

### Purpose

The purpose of this case-control study was to investigate whether chromosome aberrations (CAs) in chromosomes 1, 2 and 4 measured in apparently healthy cells (PBL) using a fluorescence *in situ* hybridization (FISH) technique will help to predict the odds of developing CRC. As dietary habits are known to be one of the main determinants of sporadic CRC, fruit and vegetable consumption has been considered as a main confounding variable in this relationship.

## Materials and Methods

### Study design and sample

A hospital based case-control study included CRC patients admitted to the I Chair of General Surgery and Department of Gastroenterological Surgery, Jagiellonian University—Medical College, Krakow, Poland, and controls treated due to other causes (unrelated to cancer) at the University Hospital in Krakow. The design of the study has been described elsewhere [12,13]. In brief, participants were newly diagnosed cases of sporadic adenocarcinoma of either the colon or rectum and were confirmed histopathologically. Inclusion criteria were: age of up to 75 years, Caucasian, being a native of Poland, referred for a surgical treatment of CRC (cancer cases) or for a treatment of a condition unrelated to cancer (controls). Next the following exclusion criteria were implemented: radio- or chemotherapy before taking a blood sample, presence of communication (verbal contact) problems and/or cognitive limitations, diagnosis of secondary cancer localized in colon or rectum (i.e., distant metastasis in large bowel), diagnosis of primary cancer other than colorectal, recurrent cancer, underwent surgery (before recruitment) of gastrointestinal tract, present or past diagnosis of chronic disease of gastrointestinal tract (diverticulitis, irritable bowel syndrome, acute or chronic gastric ulcer, acute/chronic pancreatitis), diabetes (any type), renal failure, and hepatic insufficiency. Additionally, subjects reporting to have gastrointestinal symptoms in a period of 5-years before an interview were also excluded.

The primary case-control study was planned to have 80 subjects in total for genetic investigation and was planned to use a matching strategy (cases and controls were matched according to gender and age +/- 5 years). Upon entrance into the study, subjects were asked to participate in the genetic part of the project. After written informed consent was obtained, an interview was performed and blood samples were taken. Out of 80 subjects 2 were excluded due to exclusion criteria which were revealed after the blood sample was taken (cancer diagnosis in the past, living abroad in childhood). According to the protocol every patient was asked to provide two blood samples for classical chromosome aberration assessment, two for sister chromatid exchanges (SCE) and additionally four samples for fluorescence *in situ* hybridization (two for the assessment of the current status, and two for irradiation). In approximately half of the patients collected blood samples were not sufficient (due to limited volume) for all planned genetic testing. Results of chromosome aberrations assessed by the classical cytogenetic method were published in the primary study monograph [14]. Results of the SCE assessment from the next set of 38 subjects, which included a set of samples from another 24 subjects coming from a similar research project were also published [12]. Finally, another set of 38 blood samples (20 colon cancer cases and 18 controls) were also available for the FISH technique to evaluate the presence and frequency of chromosome 1, 2 and 4 aberrations. Although inclusion criteria for the primary study covered colon and rectal cancer cases, only colon (ICD-X: C18) cancer cases participated in the FISH part. Entire study was conducted in accordance with the ethical principles of the Declaration of Helsinki and was approved by the Bioethical Committee of Jagiellonian University (number KBET/115/B/2011).

### Tools and data collection

Study participants were interviewed about their dietary habits, demographic characteristics, and other potential covariates. Clinical information was collected from the hospital medical records. Dietary habits were assessed by a semi-quantitative food frequency questionnaire (SFFQ). In total, 148-dietary items were used including questions about consumption of fresh fruits (summer/winter time), salads and fresh and cooked vegetables, and for each food or beverage item, a commonly consumed portion size was specified by standardized photographs. Trained interviewers gathered information about usual (habitual) consumption over the period of 1 year by calendar seasons. In order to assess habitual dietary patterns, CRC cases were asked about their dietary habits at a time of 5 years prior to the onset of gastrointestinal symptoms (if present) or prior to the beginning of the diagnostic process. The validity and reproducibility of the questionnaire was assessed and published elsewhere [15].

### Cytogenetic analysis

#### Blood collection and metaphase spread preparation

Blood samples were collected by phlebotomy into lithium-heparinized vacutainers, coded, and then quickly transported to the laboratory in the H. Niewodniczanski Institute of Nuclear Physics Polish Academy of Sciences in Krakow, Poland (IFJ PAN) before any medical intervention. Briefly, whole blood (1,6 ml) was added to 15 ml of RPMI 1640 culture medium supplemented with 20% heat-inactivated fetal bovine serum, L-glutamine (2 mM) and antibiotics (100 U/ml penicillin and 100 g/ml streptomycin). Lymphocytes were stimulated by phytohaemagglutinin (PHA) and cultured for 72 hours, 37°C at 5% CO_2_ in a humidified incubator. Two hours before the end of culturing, 200 μl of colcemid solution (to stop dividing cells in metaphase) was added. Next, cells were treated with hypotonic KCl and fixed in 3:1 methanol/acetic acid. Metaphase spreads were prepared by dropping the fixed cells (1–2 drops) on clean slides. Slides were then dried at room temperature (RT) and stored in a -20°C freezer.

#### Staining

The details of the FISH method are described elsewhere [16]. Briefly, staining was performed according to the protocol provided by a representative of the manufacturer of the probes (Cytocell LPP124, Whole Chromosome Paint Combination 1, 2, 4). Slides were dried at RT for 15–20 minutes and checked for adequate chromosome spreading to determine the suitability of specimen for the *in situ* hybridization procedure. The slides were soaked in saline-sodium citrate (SSC) for 2 minutes at 37°C, incubated with Pepsin Solution at RT for 5 minutes and were put into phosphate buffered saline (PBS) at RT for 3 minutes. Slides were dehydrated in a series of ethanol baths (70%, 90%, 100%) at RT for 1 minute each and dried for 10–15 minutes at RT. Then 10 μl of probe (warmed to RT) was placed on the slide and cover-slipped with a 22x22 mm glass cover slip ensuring good coverage. The edges of the cover slip were sealed with glue. To denature the slides they were put into 75°C for 8 minutes. Then slides were put into 37°C for at least 24 hours for hybridization. Post-hybridization, the glue and cover slips were removed and the slides were washed in the Wash Buffer I at 72°C for 2 minutes and then quickly transferred into the Wash Buffer II for 1 minute at RT. Slides were dehydrated again in a series of ethanol baths (70%, 90%, 100%) at RT each for 1 minute and dried at RT. Next, 10 μl of 4,6-diamidino-2-phenylindole (DAPI) was applied and slides were cover-slipped and stored in dark at temperature 4 to 10°C until examination. The slides were visualized under fluorescent microscopy. The liquid probe mixture of whole chromosome painting probes 1, 2 and 4, 3 color, 3 probe combinations, Cytocell Ltd was used.

#### Scoring

**Fig 1:**
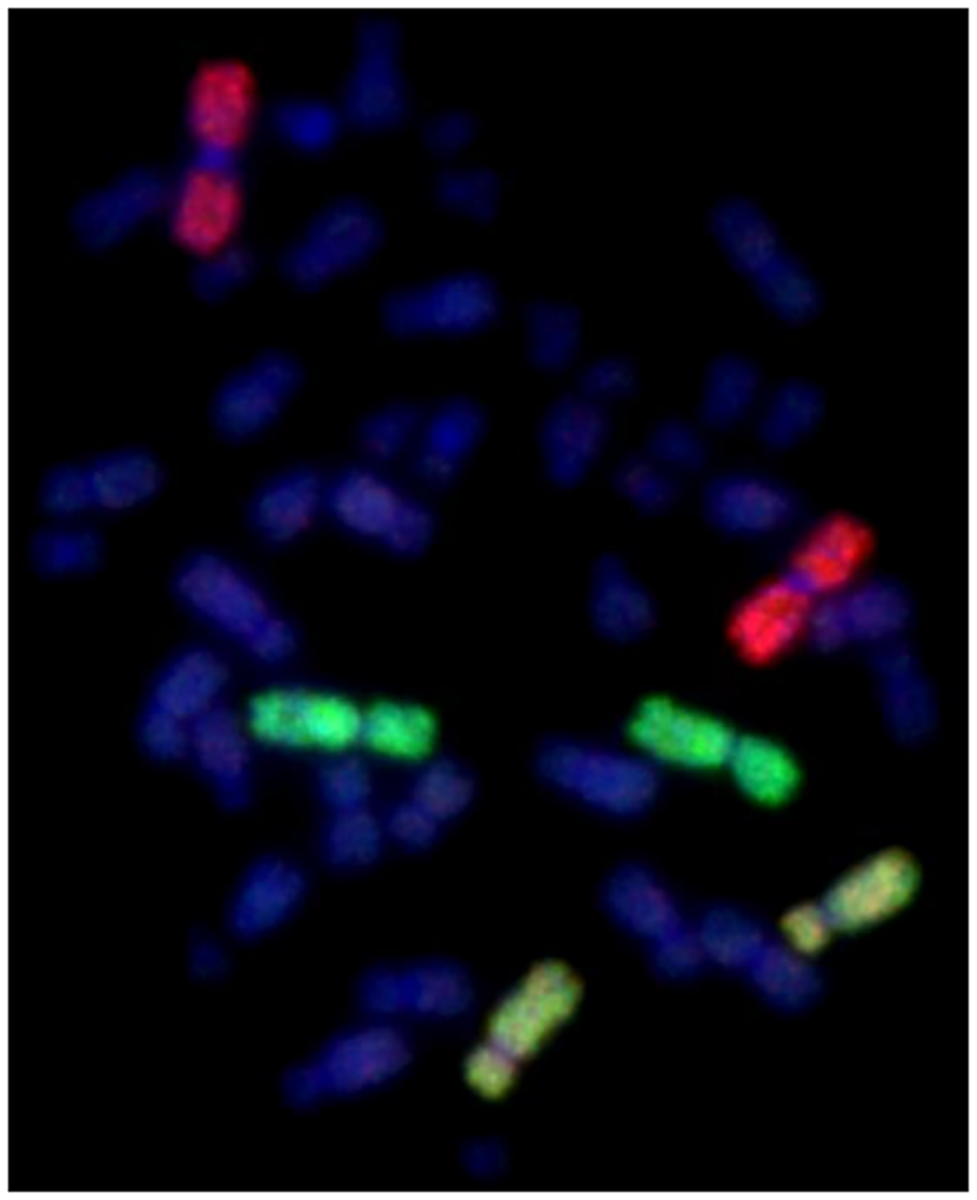
Representative example of a normal metaphase spread detected by fluorescence *in situ* hybridization (FISH) using whole chromosome paints. Chromosome pairs 1 are painted red, 2 (green) and 4 (yellow).

**Fig 2:**
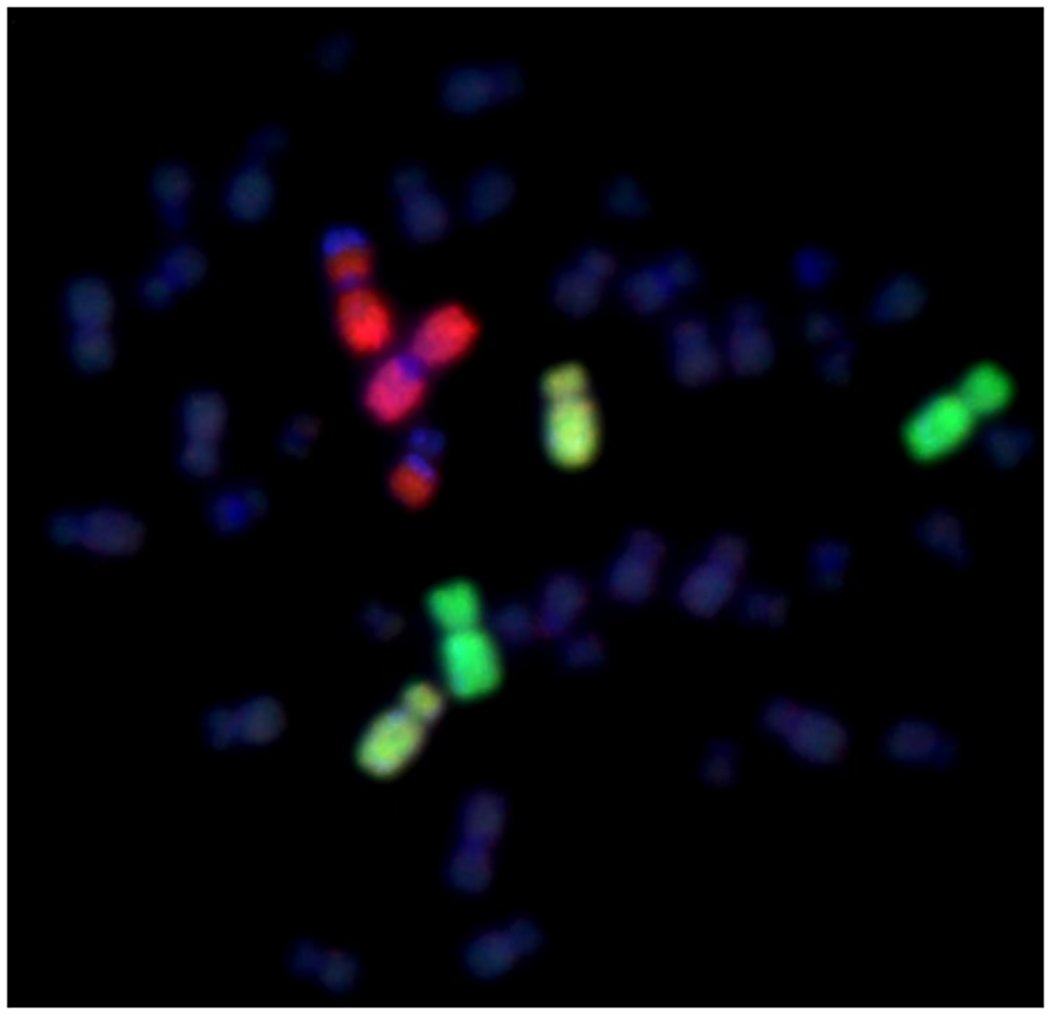
Example of a reciprocal translocation between chromosome 1 (red) and one unpainted (DAPI, blue) chromosome detected by fluorescence *in situ* hybridization (FISH) using whole chromosome paints.

Metaphase spreads were prepared from a total of 38 subjects. At least 1000 metaphases were scored for each of the 22 of study participants and for the remaining less than 1000 (4 subjects have below 500 metaphases scored) due to a low proliferation index resulting in a lower number of metaphase spreads. Metaphase spreads were scored blindly for types of damages, which included stable (translocations, deletions and insertions) (Figs 1 and 2) and unstable (acentric fragments) damage in chromosomes 1, 2, and 4. The frequency of chromosome damage was calculated and expressed for 1000 metaphases (cells). Analyses have been performed to show differences for every type of damage and for the group of stable damage as well as for the total number of aberrations.

### Statistical methods

#### Sample size considerations

We expected to observe the mean frequency of chromosome aberrations at about 3/1000 cells in the colorectal cancer and at about 1/1000 in the control group (with SD = 2/1000 cells). Assuming the alpha = 0.05 and the power goal = 0.90 we had to investigate 23 individuals per group. Assuming the power goal = 0.80 the required sample size was 17 per group. The number of blood samples was reduced as it had been primarily planned in our study, however there were still 20 and 18 blood samples available for cases and controls, respectively, therefore we believed that the sample would enable us to answer our research questions.

#### Statistics and analysis

Although the study was designed to have cases and controls matched according to age and gender, the availability of blood samples caused that the number of subjects differed across groups. Descriptive statistics have been provided by medians and limits of inter quartile range (IQR). As the sample size was relatively small and with the inability to reject the null hypothesis regarding the equivalence to normal distribution might be determined by the sample size, the differences between CRC patients and controls were tested by nonparametric U-Mann-Whitney test for continuous variables. Chi-square test was used for categorical and the Cochran-Armitage test for ordinal variables. Odds ratio (OR) obtained by logistic regression was used to assess the odds (an approximation of a risk) of CRC associated with the frequency of aberrations. The following models have been analyzed: I) a univariable model; II) a model considering dietary iron (a proxy measure of meat consumption, continuous) as a single covariate; III) a model with smoking status as a single covariate; IV) a model considering fruit and vegetable consumption (number of servings weekly, continuous) as a single covariate; and V) a model considering fruit and vegetable consumption as categorical by tertiles as a single covariate. A limited number of covariates have been used due to small sample size, but it is worth mentioning that groups were quite comparable according the basic characteristics. The analyses were done by the Stata/IC 13.1 for Windows (64-bit x86-64) StataCorp LP software. There were no missing data in the available sample. The significance level of 0.05 was used.

## Results

The basic characteristics of study participants have been presented in Table 1. The median age was 62 years (first quartile: 54, third quartile: 66), and 50% of the group was males. Controls were slightly younger, smoked more frequently, consumed more vegetables and fruits, and had a lower amount of iron in their diet. No one difference was statistically significant. CRC patients had significantly higher frequency of translocations, stable aberrations and total aberrations in chromosome 1. There were no differences observed for chromosome 2 and chromosome 4 (Table 2).

**Table 1:** Basic characteristic of study groups.

	CRC patients	Controls	p
	[n = 20]	[n = 18]	
Age [years]			
median (Q1-Q3)	62.5 (51.5–65.0)	60.0 (55.0–68.0)	p^MW^ = 0.884
Sex [n, (%)]			
males	10 (50.0%)	9 (50.0%)	df = 1, p^chi^ = 1.000
Ever smoking [n, (%)]			
Yes	7 (35.0%)	7 (38.9%)	df = 1, p^chi^ = 0.804
Vegetable and fruit consumption [servings/week]			
median (Q1-Q3)	15.1 (12.3–19.6)	16.4 (13.2–20.9)	p^MW^ = 0.492
Vegetable and fruit consumption -tertiles [servings /week]			
T1: <13.54	8 (40.0%)	5 (27.8%)	
T2: 13.54–19.43	7 (35.0%)	6 (33.3%)	df = 2,
T3: >19.43	5 (25.0%)	7 (38.9%)	p^CA^ = 0.322
Dietary iron [mg/day]			
median (Q1-Q3)	12.1 (11.1–13.7)	11.7 (10.9–14.1)	p^MW^ = 0.907
BMI [kg/m^2^]			
median (Q1-Q3)	27.1 (25.4–29.7)	27.7 (25.0–30.4)	p^MW^ = 0.629
Cancer Astler-Coller's staging [n, (%)]			
A	1 (5.0%)	---	---
B1	9 (45.0%)		
B2	2 (10.0%)		
C1	1 (5.0%)		
C2	2 (10.0%)		
D	5 (25.0%)		

CRC -colorectal cancer; T1, T2, T3—first, second and third tertile; *df*—degrees of freedom; chi—chi-square test; MW—Mann-Whitney test; CA–the Cochran Armitage test for trend

**Table 2:** Chromosome 1, 2 and 4 aberrations [/1000 cells] in study groups.

	CRC patients	Controls	p	CRC patients	Controls	p	CRC patients	Controls	p	CRC patients	Controls	p
	[n = 20]	[n = 18]		[n = 20]	[n = 18]		[n = 20]	[n = 18]		[n = 20]	[n = 18]	
	chromosome 1			chromosome 2			chromosome 4			chromosomes 1, 2, 4		
**translocations**												
n (%)	18 (90.0%)	11 (61.1%)	df = 1, p^chi^ = 0.036	14 (70.0%)	12 (66.7%)	df = 1, p^chi^ = 0.825	11 (55.0%)	6 (33.3%)	df = 1, p^chi^ = 0.180	19 (95.0%)	14 (77.8%)	df = 1, p^chi^ = 0.117
median (Q1-Q3)	3.5 (1.0–5.1)	1 (0.0–2.8)	p^MW^ = 0.006	1.9 (0.0–3.0)	1 (0.0–2.0)	p^MW^ = 0.148	1 (0.0–1.8)	0 (0.0–1.1)	p^MW^ = 0.241	6.5 (2.0–9.5)	2.5 (1.0–5.0)	p^MW^ = 0.016
**deletions**												
n (%)	2 (10.0%)	2 (11.1%)	df = 1, p^chi^ = 0.911	0 (0%)	0 (0%)	---	1 (5.0%)	2 (11.1%)	df = 1, p^chi^ = 0.485	3 (15.0%)	3 (16.7%)	df = 1, p^chi^ = 0.888
median (Q1-Q3)	0 (0–0)	0 (0–0)	p^MW^ = 0.912	0	0	---	0 (0–0)	0 (0–0)	p^MW^ = 0.491	0 (0–0)	0 (0–0)	p^MW^ = 0.835
**insertions**												
n (%)	2 (10.0%)	0 (0%)	df = 1, p^chi^ = 0.168	0 (0%)	0 (0%)	---	0 (0%)	0 (0%)	---	2 (10.0%)	0 (0%)	df = 1, p^chi^ = 0.168
median (Q1-Q3)	0 (0–0)	0	p^MW^ = 0.174	0	0	---	0	0	---	0 (0–0)	0	p^MW^ = 0.174
stable aberrations*												
n (%)	18 (90.0%)	11 (61.1%)	df = 1, p^chi^ = 0.036	14 (70.0%)	12 (66.7%)	df = 1, p^chi^ = 0.825	11 (55.0%)	6 (33.3%)	df = 1, p^chi^ = 0.180	19 (95.0%)	14 (77.8%)	df = 1, p^chi^ = 0.117
median (Q1-Q3)	3.8 (1.0–5.3)	1.0 (0.0–2.8)	p^MW^ = 0.007	1.9 (0.0–3.0)	1.0 (0.0–3.0)	p^MW^ = 0.148	1.0 (0.0–1.8)	0.0 (0.0–1.9)	p^MW^ = 0.312	7.1 (2.0–10.0)	3.0 (1.0–5.0)	p^MW^ = 0.020
**acentric fragments**												
n (%)	3 (15.0%)	2 (11.1%)	df = 1, p^chi^ = 0.723	2 (10.0%)	2 (11.1%)	df = 1, p^chi^ = 0.911	1 (5.0%)	1 (5.6%)	df = 1, p^chi^ = 0.939	5 (25.0%)	3 (16.7%)	df = 1, p^chi^ = 0.529
median (Q1-Q3)	0 (0–0)	0 (0–0)	p^MW^ = 0.691	0 (0–0)	0 (0–0)	p^MW^ = 0.956	0 (0–0)	0 (0–0)	p^MW^ = 0.940	0 (0.0–0.5)	0 (0–0)	p^MW^ = 0.525
**total aberrations**												
n (%)	18 (90.0%)	11 (61.1%)	df = 1, p^chi^ = 0.036	14 (70.0%)	11 (61.1%)	df = 1, p^chi^ = 0.564	11 (55.0%)	6 (33.3%)	df = 1, p^chi^ = 0.180	19 (95.0%)	13 (72.2%)	df = 1, p^chi^ = 0.055
median (Q1-Q3)	3.8 (1.0–5.7)	1 (0.0–3.0)	p^MW^ = 0.009	2.1 (0.0–3.0)	1 (0.0–2.0)	p^MW^ = 0.134	1 (0.0–1.8)	0 (0.0–1.9)	p^MW^ = 0.304	7.1 (2.0–11.0)	3.5 (0.0–6.0)	p^MW^ = 0.020

Q1 -first quartile; Q3 -third quartile; chi -chi-square test; MW -Mann-Whitney test, values for 1000 cells; n -represents number of subjects with chromosome aberration(s);

*-stable aberrations is the sum of translocations, deletions and insertions

As the main purpose of the study was to assess the odds of CRC associated with chromosomal damage in PBL, we have decided to perform analyses only for the chromosomes showing differences in CAs between groups. After some adjustment including also vegetable and fruit consumption, an increase in the CRC odds for translocations (OR = 1.71 to 1.82 across models), stable aberrations (OR = 1.62 to 1.75) and total aberrations (OR = 1.53 to 1.66) in chromosome 1 has been observed. The CRC risk was also increased if translocations, stable aberrations and total number of aberrations for chromosomes 1, 2 and 4 were analyzed together, but the increase in risk was modest (OR = 1.24 to 1.46) (Table 3). Additional analysis performed to assess the role of vegetable and fruit consumption revealed a decrease in the CRC risk across tertiles of consumption in the models considering either the number of stable aberrations for chromosomes 1, 2 and 4 or the total number of aberrations for these three chromosomes (Table 3). The data set underlying the findings is available in S1 File.

**Table 3:** Odds of colorectal cancer related to an increase in chromosome aberrations and to an increase in a number of fruit and vegetable servings.

	translocations -chromosome 1		stable aberrations -chromosome 1		total aberrations -chromosome 1		translocations -chromosome 1, 2, 4		stable aberrations -chromosome 1, 2, 4		total aberrations -chromosome 1, 2, 4	
	OR (95% CI)	p	OR (95% CI)	p	OR (95% CI)	p	OR (95% CI)	p	OR (95% CI)	p	OR (95% CI)	p
aberrations [OR for an increase by 1 /1000 cells]^1^	1.71 (1.13–2.57)	0.010	1.62 (1.12–2.35)	0.011	1.54 (1.09–2.17)	0.015	1.32 (1.04–1.67)	0.021	1.28 (1.03–1.58)	0.024	1.25 (1.02–1.52)	0.028
aberrations [OR for an increase by 1 /1000 cells]*	1.71 (1.13–2.57)	0.011	1.62 (1.11–2.35)	0.012	1.53 (1.09–2.16)	0.015	1.31 (1.04–1.66)	0.023	1.28 (1.03–1.58)	0.025	1.24 (1.02–1.51)	0.029
aberrations [OR for an increase by 1 /1000 cells]**	1.78 (1.16–2.72)	0.008	1.69 (1.14–2.51)	0.009	1.61 (1.12–2.31)	0.011	1.36 (1.06–1.75)	0.016	1.31 (1.05–1.64)	0.017	1.28 (1.04–1.58)	0.020
aberrations [OR for an increase by 1 /1000 cells]	1.78 (1.16–2.75)	0.009	1.69 (1.14–2.52)	0.009	1.60 (1.10–2.32)	0.013	1.41 (1.08–1.85)	0.013	1.38 (1.07–1.78)	0.014	1.34 (1.05–1.71)	0.018
vegetable and fruit consumption [OR for an increase by 1 serving /week]	0.92 (0.80–1.07)	0.275	0.92 (0.80–1.06)	0.259	0.93 (0.81–1.07)	0.289	0.89 (0.76–1.04)	0.153	0.88 (0.75–1.04)	0.134	0.89 (0.76–1.04)	0.141
total aberrations [OR for an increase by 1 /1000 cells]	1.82 (1.17–2.84)	0.008	1.75 (1.15–2.66)	0.009	1.66 (1.12–2.46)	0.012	1.46 (1.09–1.95)	0.012	1.42 (1.08–1.87)	0.013	1.40 (1.07–1.83)	0.015
vegetable and fruit consumption [servings /week]												
T1: <13.54	1 (ref.)		1 (ref.)		1 (ref.)		1 (ref.)		1 (ref.)		1 (ref.)	
T2: 13.54–19.43	0.54 (0.09–3.19)	0.496	0.49 (0.08–2.97)	0.441	0.47 (0.08–2.84)	0.411	0.29 (0.04–2.01)	0.211	0.29 (0.04–1.96)	0.203	0.29 (0.04–2.01)	0.209
T3: >19.43	0.22 (0.03–1.64)	0.140	0.21 (0.03–1.52)	0.121	0.21 (0.03–1.52)	0.123	0.14 (0.01–1.23)	0.076	0.12 (0.01–1.14)	0.065	0.11 (0.01–1.07)	0.057
p for the increase in the consumption across tertiles (trend)		0.141		0.121		0.122		0.076		0.065		0.057

^1^- univariable logistic regression;

(*)—multivariable logistic regression adjusted for dietary iron;

(**)—multivariable logistic regression adjusted for smoking status T1, T2, T3 -first, second and third tertile

## Discussion

We decided to use the FISH technique as it is perceived as a powerful tool to detect specific chromosomal abnormalities due to its high sensitivity and the speed with which the assays can be performed. The technique serves as a diagnostic tool for many cancers [17]. The FISH method is used to identify chromosome instability biomarkers in some other cancer research [18,19]. We have chosen chromosome 1, 2 and 4 because it represents 22.71% of male and 22.34% of female genome as estimated from the chromosome physical lengths [16]. There are genes as hMSH2 and hMLH1 located on the chromosome 2p and 3p [20], and MYH gene on the chromosome 1, between p32.1 and p34.3, which mutations were linked to colorectal cancer [21]. Although majority of FISH studies are performed on biopsy materials for diagnosis or classification of tumors, in presented study we used PBL for analyzing specific chromosomal aberrations involving chromosomes 1, 2, or 4.

Our study has shown an increase in aberrations in chromosome 1 as the number of translocations, the number of stable aberrations including translocations, deletions and insertions, and the total number of aberrations (stable and unstable) in PBL possibly associated with an increase in the odds of colon cancer. We didn’t observe any association for deletions or insertions for this chromosome either they were considered separately or as the number of acentric fragments. There were no differences observed in the level of damage in chromosome 2 and chromosome 4 between CRC patients and controls.

There is a general agreement that cancers, including CRC, develop as a consequence of an accumulation of genetic damage [3,22,23], therefore, measuring the frequency of chromosomal aberrations seems to be a good means for predicting the odds of cancer because of genetic polymorphisms, xenobiotic metabolism, effectiveness of DNA repair and the role of genotoxic stress. Thus, we measured the level of damage in a set of specific chromosomes in PBL. Peripheral blood can be easily collected from subjects by a simple phlebotomy and circulating lymphocytes which represent good surrogate for an assessment of chromosome alterations in tissues [24] can be easily harvested.

The frequency of chromosome aberrations in PBL has been proposed as a biomarker of genotoxicity, especially in occupational settings [25]. The relationship between high frequency of CAs and an increased cancer risk was observed in the Nordic Study and in the Italian investigation, both showing almost 2-fold increase in cancer risk in the high tertile of CAs and significant trends across tertiles [26]. The results from collaborative study of 11 different European cohorts revealed an increased cancer risk across tertiles, and additionally it was observed that the presence of ring chromosomes was associated with approximately 2-fold increase in cancer risk [27]. It should be noticed, however, that all of the aforementioned studies used CA measured by light microscopy to evaluate a general risk for all cancer types. Other studies published in that timeframe suggested that high level of CAs was more clearly associated with gastrointestinal digestive cancers, specifically stomach and CRC [28,29].

Our results suggest an increase of colon cancer odds associated with an increase in stable CAs independent of level of fruit and vegetable consumption and smoking status. This is in line with other observations that indicated an association between CAs and cancer, which was not merely due to some occupational risks or smoking status [30], suggesting that the 'quality' of genome might also play a role. There is also some evidence showing the effect of dietary compounds in preventing DNA damage [12,31,32]. As in some of our models both CAs and fruit and vegetable consumption were considered, we believe that this additional adjustment is one of the strengths in our investigation, and supports the hypothesis that increased CAs might be associated with colon cancer.

In our study we observed a decrease in colon cancer odds across tertiles of vegetable and fruit consumption and an increase associated with CAs, if they were analyzed together. This led to a question on the possible modification effect of vegetable and fruit consumption on CA and colon cancer odds. The interaction term between CAs and consumption of vegetables and fruits (the cutoff of the third tertile) was not statistically significant either for chromosome 1 translocations (p = 0.080) or for chromosome 1 total aberrations (p = 0.132) or for all chromosomes 1, 2 and 4 translocations (p = 0.174) or for all total chromosome 1, 2 and 4 aberrations (p = 0.287). Thus, our study did not clearly demonstrate a modification effect of vegetable and fruit consumption on CAs. We think, however, that it might be due to a limited sample size, and therefore this question requires further investigation.

We tested the frequency of aberrations in chromosomes 1, 2 and 4 as a predictor of odds ratio for colorectal cancer. Our results indicate that the odds of cancer is associated with the increase in the number of chromosome 1 translocations, as well as with the number of stable aberrations, and consequently a total number of chromosome 1 aberrations. We did not observe any association with chromosomes 2 or 4. There is no clear evidence of a chromosome marker for CRC risk. However, in addition to the role of chromosome 1 in CRC risk, there is also evidence showing the chromosome contains 'the most of hypermethylated genes' which might add to the risk of CRC [8]. Chromosome 1 was also suggested having CRC tumor suppressor genes [9]. Some recent studies investigating the role of chromosome losses and gains in CRC primary tumors and their synchronous metastases showed chromosomal changes (losses) for chromosomes 1, 17 and 18, and gains in chromosomes 7, 8, 13 and 20 [33]. Another study has shown gains for chromosome 1, and also for chromosomes 2, 4, 7, 8, 11 [10]. In our study we tried to assess a general susceptibility that might be 'visible' as an accumulation of damage in circulating blood lymphocytes. Considering evidence that chromosome 1 contains important genes like the *MUTYH* gene (located at 1p34.1) responsible for the DNA repair and associated with the development of familiar adenomatous polyposis as well as the *MTHFR* gene (at 1p36.3), our findings support the role of CA measurement in the prediction of CRC odds.

The purpose of our study was to investigate chromosome 1, 2 and 4 aberrations associated with colon cancer odds. Another point requiring discussion is molecular disease signature. The goal of using molecular disease signatures, as highlighted by molecular pathological epidemiology, is "to sub-classify disease to improve prediction of disease occurrence and progression for precision medicine and public health" [34]. In our study we were not able to assess genetic variants of colon cancer as the cancerous tissues were not available for us. The clinical picture of diseases suggested the FAP type of colon cancers recruited for the study.

In summary, our results show that the frequency of chromosomal aberrations, especially translocations in chromosome 1, detected in peripheral blood lymphocytes by fluorescence *in situ* hybridization whole-chromosome painting, seems to be a promising method to estimate odds ratio for CRC and may overcome limitations with conventional methods of CA analysis. Additionally, our study suggests the reasonableness of the use of biomarkers such as lymphocyte CAs in screening prevention programs for individuals at higher colon cancer risk to identify those who are at increased risk and require more frequent investigations, e.g. by sigmoidoscopy.

## Supporting Information

S1 File The data set underlying the findings in the study. (XLS)
